# Supplementary material for: Variant profiling of evolving prokaryotic populations
Source: PeerJ. 2017 Feb 16;5:e2997. doi: 10.7717/peerj.2997 (PMC5316281; doi:10.7717/peerj.2997)
Supplement: Supplemental Information 1 [file peerj-05-2997-s001.docx]

**Suppl. Table 1** Overview of organisms and datasets. The table gives an overview of the simulated and re-sequenced datasets, including genome information, variant types and counts, as well as total and variant coverages.

| Genome | G+C (%) | Size (Mb) | Variants | Type | Coverage (x) | Reads | Variant_Coverage (x) | Experiment |
| --- | --- | --- | --- | --- | --- | --- | --- | --- |
| Candidatus Protochlamydia amoebophila UWE25 | 34 | 2.4 | 135 | ALL | 400 | 9600000 | 16 | sim_135VAR |
| Candidatus Protochlamydia amoebophila UWE25 | 34 | 2.4 | 135 | ALL | 160 | 3840000 | 16 | sim_135VAR |
| Candidatus Protochlamydia amoebophila UWE25 | 34 | 2.4 | 135 | ALL | 80 | 1920000 | 16 | sim_135VAR |
| Candidatus Protochlamydia amoebophila UWE25 | 34 | 2.4 | 100 | SNP | 400 | 9600000 | 16 | sim_100SNP |
| Candidatus Protochlamydia amoebophila UWE25 | 34 | 2.4 | 100 | IndelS | 400 | 9600000 | 16 | sim_100IndS |
| Candidatus Protochlamydia amoebophila UWE25 | 34 | 2.4 | 100 | IndelL | 400 | 9600000 | 16 | sim_100IndL |
| Candidatus Protochlamydia amoebophila UWE25 | 34 | 2.4 | 50 | DUP | 400 | 9600000 | 16 | sim_50DUP |
| Candidatus Protochlamydia amoebophila UWE25 | 34 | 2.4 | 50 | ITX | 400 | 9600000 | 16 | sim_50ITX |
| Candidatus Protochlamydia amoebophila UWE25 | 34 | 2.4 | 50 | INV | 400 | 9600000 | 16 | sim_50INV |
| Streptomyces coelicolor A3 | 72 | 8.66 | 135 | ALL | 400 | 34640000 | 16 | sim_135VAR |
| Buchnera aphidicola APS | 26 | 0.68 | 135 | ALL | 400 | 2720000 | 16 | sim_135VAR |
| Rickettsia conorii Malish7 | 32 | 1.27 | 135 | ALL | 400 | 5080000 | 16 | sim_135VAR |
| Escherichia coli K12 MG1654 | 50 | 4.64 | 135 | ALL | 400 | 18560000 | 16 | sim_135VAR |
| Mycobacterium tuberculosis H37Rv | 65 | 4.41 | 135 | ALL | 400 | 17640000 | 16 | sim_135VAR |
| Sulfolobus islandicus LAL14-1 | 35 | 2.46 | 135 | ALL | 400 | 9840000 | 16 | sim_135VAR |
| Candidatus Protochlamydia amoebophila UWE25 | 34 | 2.4 | 135 | ALL | 500 | 12307692 | 20 | Simseq 500X |
| Candidatus Protochlamydia amoebophila UWE25 | 34 | 2.4 | 135 | ALL | 500 | 12307692 | 20 | pirs 500X |
| Candidatus Protochlamydia amoebophila UWE25 | 34 | 2.4 | 21000 | ALL | 400 | 9600000 | 16 | alfsim |
| Candidatus Protochlamydia amoebophila UWE25 | 34 | 2.4 | 80 | ALL | 500 | 12307692 | >10x/2%MRA | EE_reseq |

**Suppl. Table 2** Observed variant calls for synthetic variant data containing only inversions. The first column shows the inserted variant position (VARIANT_POS), the second column shown the observed caller positions for the variant (POS), the third column (INFO) shows the information about type, length, caller, read coverage and the fourth column shows reference read coverage, variant read coverage, variance of variant read coverage, total read coverage and variant read coverage in percent. Shown are the first 10 calls for in total 50 inversions. Pindel detects inversions as break positions (SVTYPE=BP) at the start and mostly also at the end of the inversion. Cortex on the other hand detects the inverted sequence itself (COMPLEX or INV_INDEL), however at lower sensitivity (e.g. 1/5 means one out of five).

| VARIANT_POS | POS | TYPE_PREDICTION | COVERAGES |
| --- | --- | --- | --- |
| 51321 | 51320 | IMPRECISE;SVTYPE=BP;SVLEN=non;SVCALL=pindel_B,4/5,BP,non,nd/5 | nd,5:0:205:2 |
| 51321 | 52629 | IMPRECISE;SVTYPE=BP;SVLEN=non;SVCALL=pindel_B,3/5,BP,non,nd/8 | nd,8:0:202:3 |
| 64717 | 64718 | IMPRECISE;SVTYPE=BP;SVLEN=non;SVCALL=pindel_B,3/5,BP,non,nd/6 | nd,6:0:217:2 |
| 64717 | 65826 | IMPRECISE;SVTYPE=BP;SVLEN=non;SVCALL=pindel_B,4/5,BP,non,nd/5 | nd,5:0:184:2 |
| 126696 | 126696 | SVTYPE=COMPLEX;SVLEN=0;SVCALL=cortex,1/5,COMPLEX,0,1554/275 | 1554,275:0:173:158 |
| 126696 | 126696 | IMPRECISE;SVTYPE=BP;SVLEN=non;SVCALL=pindel_B,5/5,BP,non,nd/6 | nd,6:0:173:3 |
| 126696 | 128532 | IMPRECISE;SVTYPE=BP;SVLEN=non;SVCALL=pindel_B,2/5,BP,non,nd/6 | nd,6:0:188:3 |
| 221395 | 222348 | IMPRECISE;SVTYPE=BP;SVLEN=non;SVCALL=pindel_B,3/5,BP,non,nd/6 | nd,6:0:188:3 |
| 251862 | 251861 | IMPRECISE;SVTYPE=BP;SVLEN=non;SVCALL=pindel_B,2/5,BP,non,nd/4 | nd,4:0:190:2 |
| 251862 | 252969 | IMPRECISE;SVTYPE=BP;SVLEN=non;SVCALL=pindel_B,2/5,BP,non,nd/6 | nd,6:0:212:2 |
| 267649 | 267648 | SVTYPE=COMPLEX;SVLEN=0;SVCALL=cortex,1/5,COMPLEX,0,1000/187 | 1000,187:0:192:97 |
| 267649 | 267648 | IMPRECISE;SVTYPE=BP;SVLEN=non;SVCALL=pindel_B,2/5,BP,non,nd/4 | nd,4:0:192:2 |
| 267649 | 268801 | IMPRECISE;SVTYPE=BP;SVLEN=non;SVCALL=pindel_B,4/5,BP,non,nd/6 | nd,6:0:193:3 |
| 371674 | 371673 | IMPRECISE;SVTYPE=BP;SVLEN=non;SVCALL=pindel_B,3/5,BP,non,nd/5 | nd,5:0:186:2 |
| 371674 | 372470 | IMPRECISE;SVTYPE=BP;SVLEN=non;SVCALL=pindel_B,5/5,BP,non,nd/6 | nd,6:0:197:3 |
| 399846 | 399845 | IMPRECISE;SVTYPE=BP;SVLEN=non;SVCALL=pindel_B,2/5,BP,non,nd/6 | nd,6:0:197:3 |
| 399846 | 401820 | IMPRECISE;SVTYPE=BP;SVLEN=non;SVCALL=pindel_B,5/5,BP,non,nd/5 | nd,5:0:203:2 |
| 431218 | 431217 | IMPRECISE;SVTYPE=BP;SVLEN=non;SVCALL=pindel_B,3/5,BP,non,nd/6 | nd,6:0:203:2 |
| 431218 | 432414 | IMPRECISE;SVTYPE=BP;SVLEN=non;SVCALL=pindel_B,4/5,BP,non,nd/5 | nd,5:0:177:2 |
| 481825 | 481825 | SVTYPE=COMPLEX;SVLEN=0;SVCALL=cortex,1/5,COMPLEX,0,401/59 | 401,59:0:191:30 |
| 481825 | 481825 | IMPRECISE;SVTYPE=BP;SVLEN=non;SVCALL=pindel_B,3/5,BP,non,nd/6 | nd,6:0:191:3 |
| 481825 | 482218 | IMPRECISE;SVTYPE=BP;SVLEN=non;SVCALL=pindel_B,5/5,BP,non,nd/7 | nd,7:0:212:3 |
| 609994 | 609993 | IMPRECISE;SVTYPE=BP;SVLEN=non;SVCALL=pindel_B,2/5,BP,non,nd/9 | nd,9:0:185:4 |
| 609994 | 610981 | IMPRECISE;SVTYPE=BP;SVLEN=non;SVCALL=pindel_B,1/5,BP,non,nd/4 | nd,4:0:183:2 |
| 641028 | 641027 | IMPRECISE;SVTYPE=BP;SVLEN=non;SVCALL=pindel_B,3/5,BP,non,nd/6 | nd,6:0:213:2 |
| 641028 | 642370 | IMPRECISE;SVTYPE=BP;SVLEN=non;SVCALL=pindel_B,2/5,BP,non,nd/5 | nd,5:0:207:2 |
| 650739 | 650738 | IMPRECISE;SVTYPE=BP;SVLEN=non;SVCALL=pindel_B,2/5,BP,non,nd/6 | nd,6:0:184:3 |
| 650739 | 651513 | IMPRECISE;SVTYPE=BP;SVLEN=non;SVCALL=pindel_B,3/5,BP,non,nd/5 | nd,5:0:197:2 |
| 658882 | 658881 | SVTYPE=COMPLEX;SVLEN=0;SVCALL=cortex,1/5,COMPLEX,0,502/85 | 502,85:0:179:47 |
| 658882 | 658881 | IMPRECISE;SVTYPE=BP;SVLEN=non;SVCALL=pindel_B,3/5,BP,non,nd/6 | nd,6:0:179:3 |
| 658882 | 659403 | IMPRECISE;SVTYPE=BP;SVLEN=non;SVCALL=pindel_B,2/5,BP,non,nd/6 | nd,6:0:187:3 |
| 680013 | 680012 | IMPRECISE;SVTYPE=BP;SVLEN=non;SVCALL=pindel_B,3/5,BP,non,nd/4 | nd,4:0:190:2 |
| 680013 | 681743 | IMPRECISE;SVTYPE=BP;SVLEN=non;SVCALL=pindel_B,4/5,BP,non,nd/6 | nd,6:0:200:3 |
| 687351 | 687352 | IMPRECISE;SVTYPE=BP;SVLEN=non;SVCALL=pindel_B,2/5,BP,non,nd/8 | nd,8:0:201:3 |
| 834040 | 834595 | IMPRECISE;SVTYPE=BP;SVLEN=non;SVCALL=pindel_B,1/5,BP,non,nd/6 | nd,6:0:192:3 |
| 919055 | 920874 | IMPRECISE;SVTYPE=BP;SVLEN=non;SVCALL=pindel_B,4/5,BP,non,nd/5 | nd,5:0:207:2 |
| 942949 | 942948 | IMPRECISE;SVTYPE=BP;SVLEN=non;SVCALL=pindel_B,3/5,BP,non,nd/6 | nd,6:0:190:3 |
| 942949 | 944014 | IMPRECISE;SVTYPE=BP;SVLEN=non;SVCALL=pindel_B,2/5,BP,non,nd/5 | nd,5:0:207:2 |
| 958460 | 958459 | IMPRECISE;SVTYPE=BP;SVLEN=non;SVCALL=pindel_B,3/5,BP,non,nd/8 | nd,8:0:200:4 |
| 958460 | 960220 | IMPRECISE;SVTYPE=BP;SVLEN=non;SVCALL=pindel_B,3/5,BP,non,nd/5 | nd,5:0:187:2 |
| 967751 | 967750 | SVTYPE=COMPLEX;SVLEN=0;SVCALL=cortex,1/5,COMPLEX,0,704/106 | 704,106:0:175:60 |
| 967751 | 967750 | IMPRECISE;SVTYPE=BP;SVLEN=non;SVCALL=pindel_B,2/5,BP,non,nd/4 | nd,4:0:175:2 |
| 967751 | 968529 | IMPRECISE;SVTYPE=BP;SVLEN=non;SVCALL=pindel_B,2/5,BP,non,nd/5 | nd,5:0:187:2 |
| 1043227 | 1043226 | IMPRECISE;SVTYPE=BP;SVLEN=non;SVCALL=pindel_B,1/5,BP,non,nd/4 | nd,4:0:198:2 |
| 1043227 | 1045117 | IMPRECISE;SVTYPE=BP;SVLEN=non;SVCALL=pindel_B,4/5,BP,non,nd/6 | nd,6:0:180:3 |
| 1050625 | 1052241 | IMPRECISE;SVTYPE=BP;SVLEN=non;SVCALL=pindel_B,4/5,BP,non,nd/5 | nd,5:0:204:2 |
| 1071667 | 1073009 | IMPRECISE;SVTYPE=BP;SVLEN=non;SVCALL=pindel_B,2/5,BP,non,nd/4 | nd,4:0:177:2 |
| 1089367 | 1089366 | SVTYPE=INV_INDEL;SVLEN=0;SVCALL=cortex,2/5,INV_INDEL,0,577/75 | 577,75:0:211:35 |
| 1089367 | 1089366 | IMPRECISE;SVTYPE=BP;SVLEN=non;SVCALL=pindel_B,3/5,BP,non,nd/6 | nd,6:0:211:2 |
| 1089367 | 1089932 | IMPRECISE;SVTYPE=BP;SVLEN=non;SVCALL=pindel_B,2/5,BP,non,nd/6 | nd,6:0:183:3 |

**Suppl. Table 3** Annotations of SNPs and small Indels by SNPEff. The EFF tag at the end of each line includes the descriptions about the implications of the variant (e.g. NON-SYNONYMOUS, INTERGENIC).

| **Position** | **Ref** | **Alt** | **Classification** |
| --- | --- | --- | --- |
| 40814 | G | A | SVTYPE=SNP;EFF=NON_SYNONYMOUS_CODING(MODERATE\|MISSENSE\|aCt/aTt\|T418I\|1511\|pc0026\|\|CODING\|pc0026\|) |
| 138004 | G | A | SVTYPE=SNP;EFF=SYNONYMOUS_CODING(LOW\|SILENT\|gtG/gtA\|V624\|729\|copA\|\|CODING\|pc0078\|) |
| 232175 | G | A | SVTYPE=SNP;EFF=NON_SYNONYMOUS_CODING(MODERATE\|MISSENSE\|Gca/Aca\|A203T\|299\|pc0154\|\|CODING\|pc0154\|) |
| 294277 | G | A | SVTYPE=SNP;EFF=NON_SYNONYMOUS_CODING(MODERATE\|MISSENSE\|gGc/gAc\|G69D\|337\|ppaA\|\|CODING\|pc0199\|) |
| 296727 | TC | T | SVTYPE=DEL/IND;EFF=FRAME_SHIFT(HIGH\|\|-\|-67\|78\|sctS\|\|CODING\|pc0201\|1) |
| 309005 | GA | G | SVTYPE=DEL/IND;EFF=FRAME_SHIFT(HIGH\|\|-\|-327\|671\|tsp\|\|CODING\|pc0214\|1) |
| 441769 | C | T | SVTYPE=SNP;EFF=NON_SYNONYMOUS_CODING(MODERATE\|MISSENSE\|Ggc/Agc\|G85S\|602\|lepA\|\|CODING\|pc0322\|) |
| 483734 | G | A | SVTYPE=SNP;EFF=NON_SYNONYMOUS_CODING(MODERATE\|MISSENSE\|Gtt/Att\|V62I\|82\|pc0357\|\|CODING\|pc0357\|) |
| 556945 | G | A | SVTYPE=SNP;EFF=NON_SYNONYMOUS_CODING(MODERATE\|MISSENSE\|Gat/Aat\|D98N\|167\|rpsE\|\|CODING\|pc0428\|) |
| 611482 | A | G | SVTYPE=SNP;EFF=NON_SYNONYMOUS_CODING(MODERATE\|MISSENSE\|tAt/tGt\|Y148C\|592\|ptsI\|\|CODING\|pc0476\|) |
| 641192 | G | A | SVTYPE=SNP;EFF=INTERGENIC(MODIFIER\|\|\|\|\|\|\|\|\|) |
| 641212 | C | T | SVTYPE=SNP;EFF=INTERGENIC(MODIFIER\|\|\|\|\|\|\|\|\|) |
| 648389 | G | T | SVTYPE=SNP;EFF=NON_SYNONYMOUS_CODING(MODERATE\|MISSENSE\|gGg/gTg\|G507V\|541\|pc0514\|\|CODING\|pc0514\|) |
| 648392 | C | T | SVTYPE=SNP;EFF=NON_SYNONYMOUS_CODING(MODERATE\|MISSENSE\|gCc/gTc\|A508V\|541\|pc0514\|\|CODING\|pc0514\|) |
| 688460 | G | A | SVTYPE=SNP;EFF=NON_SYNONYMOUS_CODING(MODERATE\|MISSENSE\|aCc/aTc\|T133I\|245\|pc0553\|\|CODING\|pc0553\|) |
| 704914 | A | C | SVTYPE=SNP;EFF=NON_SYNONYMOUS_CODING(MODERATE\|MISSENSE\|Aat/Cat\|N101H\|481\|nuoM\|\|CODING\|pc0571\|) |
| 732643 | A | G | SVTYPE=SNP;EFF=NON_SYNONYMOUS_CODING(MODERATE\|MISSENSE\|Aaa/Gaa\|K56E\|89\|infA\|\|CODING\|pc0594\|) |
| 735008 | T | TA | SVTYPE=INS/IND;EFF=FRAME_SHIFT(HIGH\|\|-/A\|-48?\|67\|nusG\|\|CODING\|pc0598\|1) |
| 744720 | C | T | SVTYPE=SNP;EFF=NON_SYNONYMOUS_CODING(MODERATE\|MISSENSE\|cCa/cTa\|P849L\|1388\|rpoC\|\|CODING\|pc0605\|) |
| 745058 | C | T | SVTYPE=SNP;EFF=NON_SYNONYMOUS_CODING(MODERATE\|MISSENSE\|Cgt/Tgt\|R962C\|1388\|rpoC\|\|CODING\|pc0605\|) |
| 845218 | A | G | SVTYPE=SNP;EFF=SYNONYMOUS_CODING(LOW\|SILENT\|ttT/ttC\|F285\|1017\|glyS\|\|CODING\|pc0693\|) |
| 867911 | C | T | SVTYPE=SNP;EFF=NON_SYNONYMOUS_CODING(MODERATE\|MISSENSE\|Gct/Act\|A422T\|445\|pc0714\|\|CODING\|pc0714\|) |
| 867936 | G | A | SVTYPE=SNP;EFF=SYNONYMOUS_CODING(LOW\|SILENT\|ggC/ggT\|G413\|445\|pc0714\|\|CODING\|pc0714\|) |
| 1089984 | GA | G | SVTYPE=DEL/IND;EFF=FRAME_SHIFT(HIGH\|\|-\|-410\|801\|pc0899\|\|CODING\|pc0899\|1) |
| 1149395 | TA | T | SVTYPE=DEL/IND;EFF=FRAME_SHIFT(HIGH\|\|-\|-43\|530\|lig\|\|CODING\|pc0950\|1) |
| 1203101 | AT | A | SVTYPE=DEL/IND;EFF=FRAME_SHIFT(HIGH\|\|-\|-165\|936\|pc1009\|\|CODING\|pc1009\|1) |
| 1230711 | T | C | SVTYPE=SNP;EFF=INTERGENIC(MODIFIER\|\|\|\|\|\|\|\|\|) |
| 1256060 | C | T | SVTYPE=SNP;EFF=NON_SYNONYMOUS_CODING(MODERATE\|MISSENSE\|tCt/tTt\|S364F\|457\|pc1055\|\|CODING\|pc1055\|) |
| 1296033 | G | A | SVTYPE=SNP;EFF=NON_SYNONYMOUS_CODING(MODERATE\|MISSENSE\|atG/atA\|M198I\|451\|dnaA\|\|CODING\|pc1082\|) |
| 1338568 | AT | A | SVTYPE=DEL/IND;EFF=FRAME_SHIFT(HIGH\|\|-\|-633\|682\|pc1117\|\|CODING\|pc1117\|1) |
| 1339224 | AT | A | SVTYPE=DEL/IND;EFF=FRAME_SHIFT(HIGH\|\|-\|-414\|682\|pc1117\|\|CODING\|pc1117\|1) |
| 1339720 | A | T | SVTYPE=SNP;EFF=STOP_GAINED(HIGH\|NONSENSE\|tTa/tAa\|L249*\|682\|pc1117\|\|CODING\|pc1117\|) |
| 1466036 | G | G,GA | SVTYPE=DEL/INS/IND;EFF=FRAME_SHIFT(HIGH\|\|-/A\|-82?\|354\|pc1231\|\|CODING\|pc1231\|1) |
| 1523646 | G | A | SVTYPE=SNP;EFF=SYNONYMOUS_CODING(LOW\|SILENT\|ttG/ttA\|L468\|618\|pc1272\|\|CODING\|pc1272\|) |
| 1603884 | CT | C | SVTYPE=DEL/IND;EFF=INTERGENIC(MODIFIER\|\|\|\|\|\|\|\|\|),UPSTREAM(MODIFIER\|\|\|\|489\|ntt_5\|\|CODING\|pc1343\|) |
| 1691317 | C | T | SVTYPE=SNP;EFF=INTERGENIC(MODIFIER\|\|\|\|\|\|\|\|\|) |
| 1726895 | G | A | SVTYPE=SNP;EFF=SYNONYMOUS_CODING(LOW\|SILENT\|agC/agT\|S1663\|1866\|pc1455\|\|CODING\|pc1455\|) |
| 1873023 | G | A | SVTYPE=SNP;EFF=SYNONYMOUS_CODING(LOW\|SILENT\|Cta/Tta\|L252\|380\|pc1563\|\|CODING\|pc1563\|) |
| 2049889 | G | A | SVTYPE=SNP;EFF=NON_SYNONYMOUS_CODING(MODERATE\|MISSENSE\|Gaa/Aaa\|E345K\|364\|pc1710\|\|CODING\|pc1710\|) |
| 2085026 | N | G | SVTYPE=SNP;EFF=INTERGENIC(MODIFIER\|\|\|\|\|\|\|\|\|) |
| 2131046 | A | G | SVTYPE=SNP;EFF=INTERGENIC(MODIFIER\|\|\|\|\|\|\|\|\|) |
| 2160129 | CA | C | SVTYPE=DEL/IND;EFF=FRAME_SHIFT(HIGH\|\|-\|-339\|351\|znuB\|\|CODING\|pc1807\|1) |
| 2278245 | G | A | SVTYPE=SNP;EFF=NON_SYNONYMOUS_CODING(MODERATE\|MISSENSE\|cGt/cAt\|R544H\|683\|pc1915\|\|CODING\|pc1915\|) |
| 2301994 | C | T | SVTYPE=SNP;EFF=NON_SYNONYMOUS_CODING(MODERATE\|MISSENSE\|Ggc/Agc\|G388S\|504\|pc1935\|\|CODING\|pc1935\|) |
| 2375412 | T | TTA | SVTYPE=IND;EFF=FRAME_SHIFT(HIGH\|\|-/TA\|-488?\|537\|pc1992\|\|CODING\|pc1992\|1) |
| 2406491 | AT | A | SVTYPE=DEL/IND;EFF=INTERGENIC(MODIFIER\|\|\|\|\|\|\|\|\|) |
| 2411844 | G | A | SVTYPE=SNP;EFF=STOP_GAINED(HIGH\|NONSENSE\|Caa/Taa\|Q10*\|426\|pc2027\|\|CODING\|pc2027\|) |

**Suppl. Table 4** Possible false positives within homopolymer regions. Short InDel positions at low frequencies, which are located within homopolymer regions (hp_length denotes the length of the homopolymer stretch) and were found within a long-term cultivation experiment. These variant positions were flagged as possible FP.

| **Position** | **Ref** | **Alt** | **Info** | **Frequency** | **hp_length** | **hp_base** |
| --- | --- | --- | --- | --- | --- | --- |
| 309005 | GA | G | SVTYPE=DEL/IND;SVLEN=-1 | 2 | 10 | A |
| 1149395 | TA | T | SVTYPE=DEL/IND;SVLEN=-1 | 2 | 10 | A |
| 1466036 | G | G,GA | SVTYPE=DEL/INS/IND;SVLEN=-1 | 3 | 12 | A |
| 1603884 | CT | C | SVTYPE=DEL/IND;SVLEN=-1 | 2 | 10 | T |

**
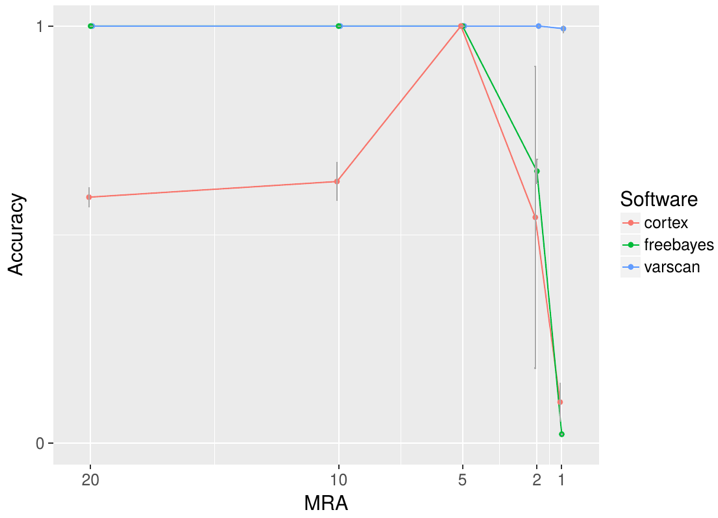
**

**Suppl. Figure 1** Accuracy of the different variant calling tools. We simulated the minor allele frequencies at 40, 20, 10 and 4 percent and used MRA cutoff values of 20, 10, 5, 2 and 1 percent. We visualized only Cortex_var and Varscan2 as they were the only tools that produced false positives after applying the MRA filters. While the accuracy for varscan decreased at one percent, cortex showed an intermediate peak at 5 percent. This is due to the fact, that cortex, using an assembly method, is more sensitive to coverage. The increased coverage increased the sensitivity (more TP, less FP), however, at a coverage above 400x (used at MRA 2) the increased prediction of FP start to lower the accuracy.


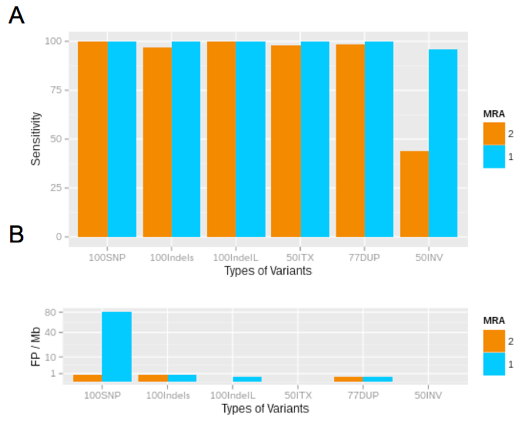


**Suppl. Figure 2** Variant detection rate of subpopulations that harbour one type of variant exclusively according to different types of variants which were inserted separately into the *P. amoebophila* reference genome. **A** shows the detection rate of true positives in % at a MRA of 2% and MRA of 1%. **B** indicates false positives per Megabase (MRA 2% , MRA 1%). The total coverage is at 400x, the coverage of the subpopulations containing either 100 or 77 or 50 variants is at 16x.

SNP Single nucleotide polymorphism

IndelS Short InDels (<10nt)

IndelL Large InDels (>=10nt)

ITX Intrachromosomal Translocation

DUP Duplication

INV Inversion


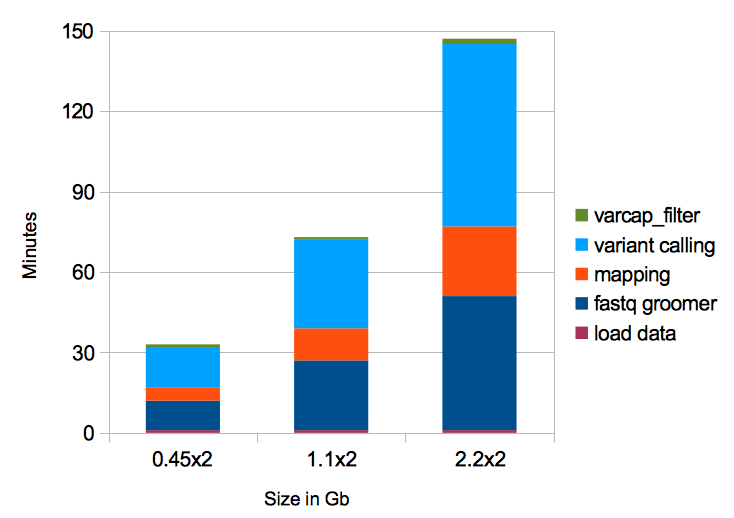


**Suppl. Figure 3** VarCap runtime in Galaxy depending on data size. The stacked barchart shows the total runtime of the Galaxy instance of VarCap as well as the major underlying modules on an 8 core/32Gb RAM virtual machine.  The most time is spent by quality filtering (fastq groomer), mapping and variant calling.

**Suppl. File1 and Suppl. File2** Raw alignment files for Sanger sequencing of the positions 1339224, 1339720, 1338568, which were present as subpopulations at 4, 11 and 28%. The alignments are provided as files: suppl_file1.txt and suppl_file2.txt
