# Supplementary figures and images for: Variant profiling of evolving prokaryotic populations
Source: PeerJ. 2017 Feb 16;5:e2997. doi: 10.7717/peerj.2997 (PMC5316281; doi:10.7717/peerj.2997)

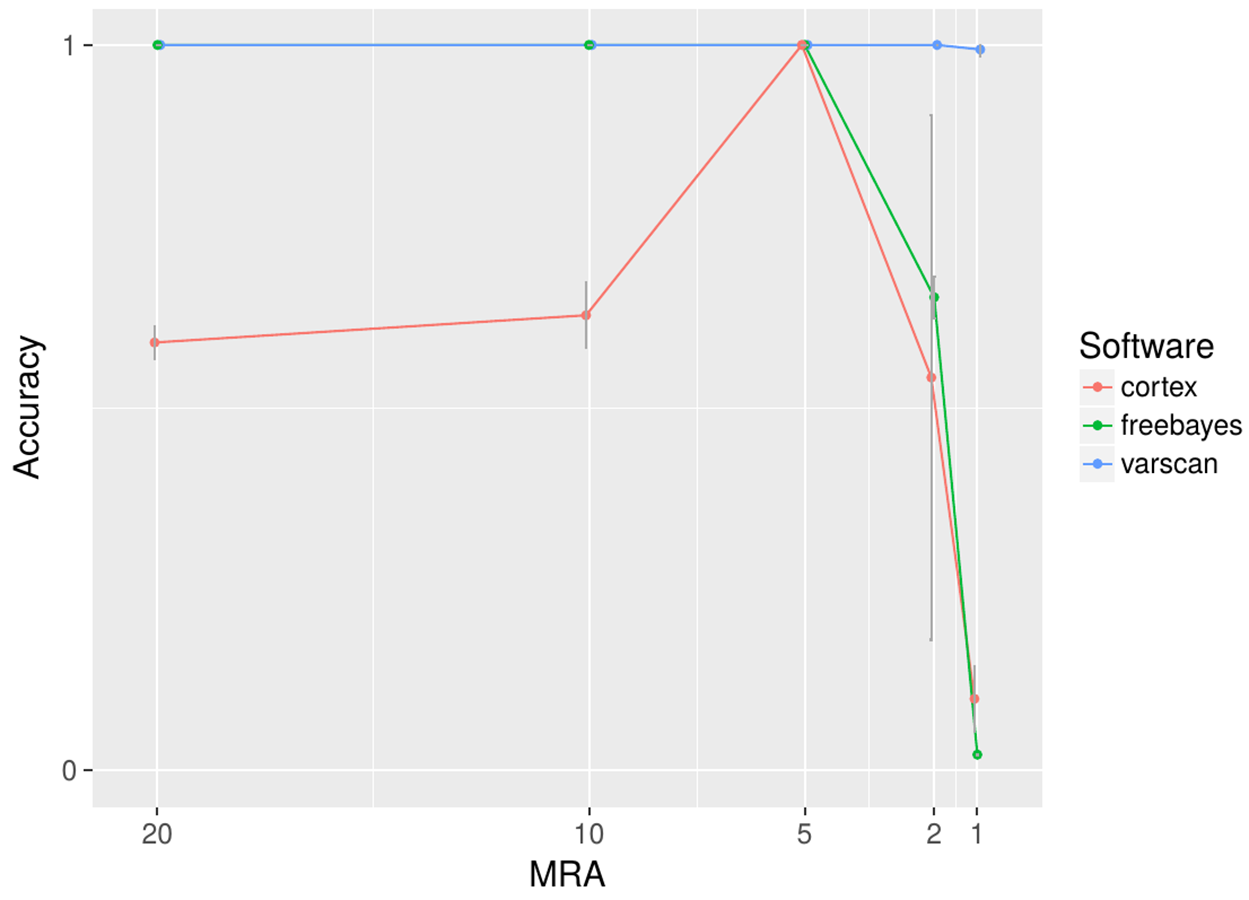

Supplement: Supplemental Information 2 [file peerj-05-2997-s002.png]

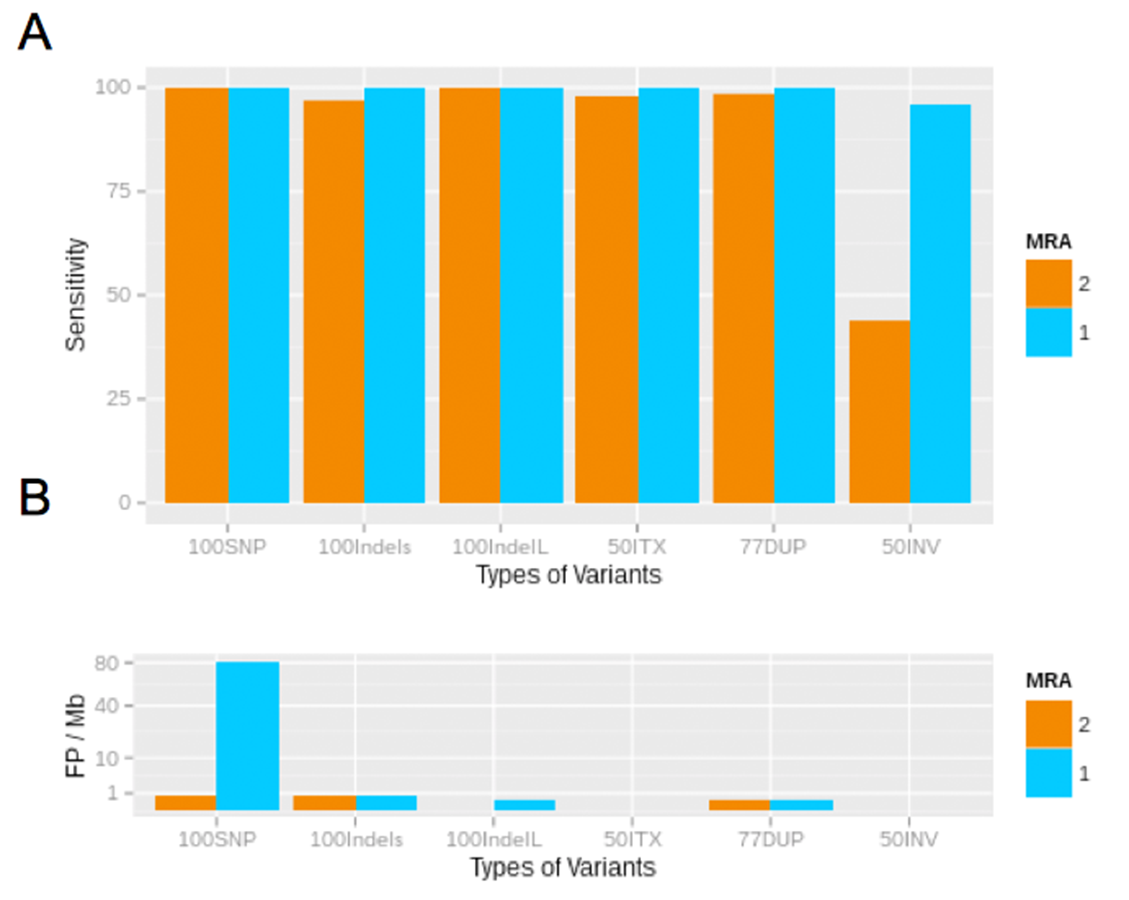

Supplement: Supplemental Information 3 [file peerj-05-2997-s003.png]

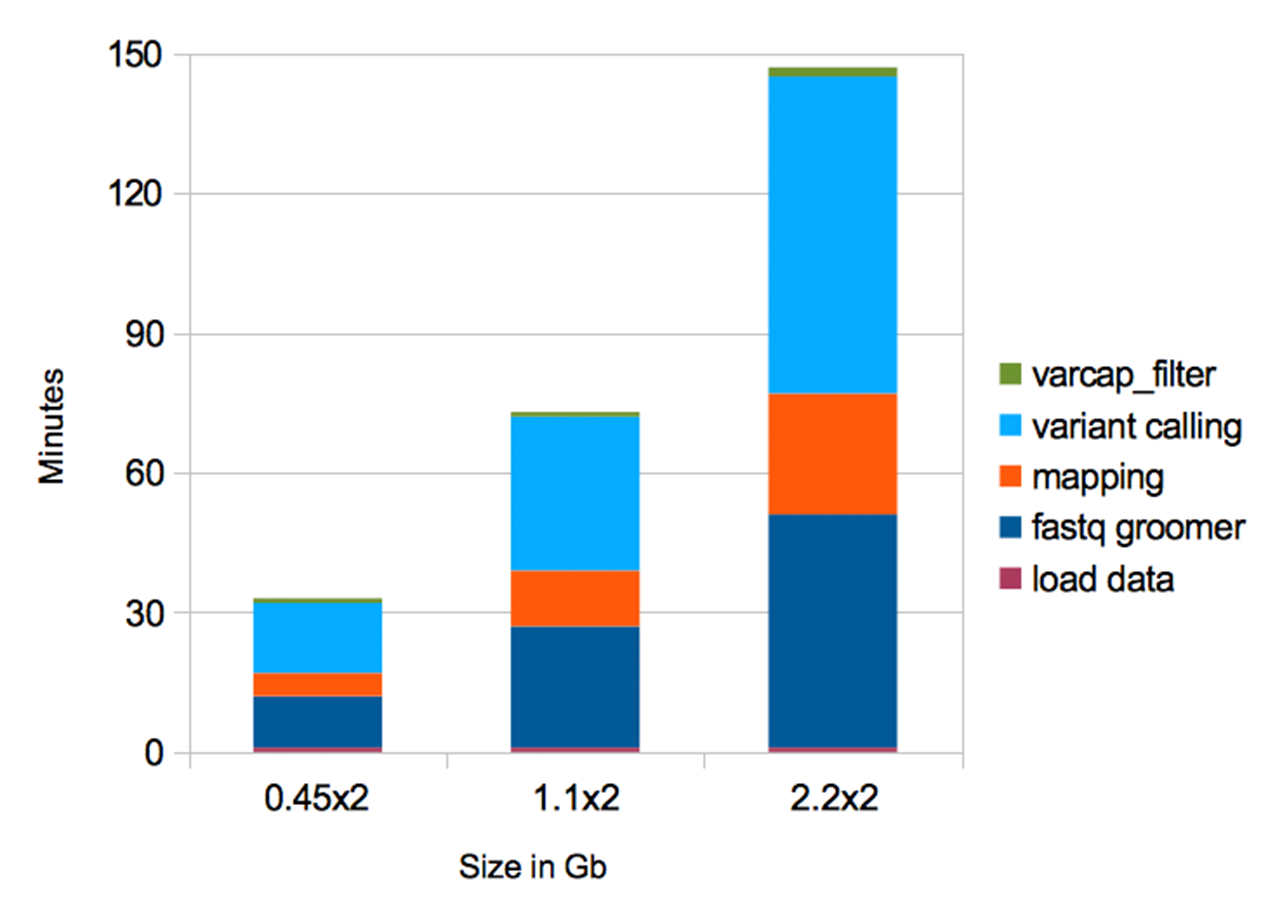

Supplement: Supplemental Information 4 [file peerj-05-2997-s004.png]
